# Anti-PL-12 and Interstitial Lung Disease as the First Signs of Antisynthetase Syndrome in Patients With Previously Diagnosed Systemic Lupus Erythematosus

**DOI:** 10.7759/cureus.93472

**Published:** 2025-09-29

**Authors:** Ivana Djuran, Bojana Ljubicic, Ana Lazarevic, Milica Popovic, Violeta Knezevic

**Affiliations:** 1 Nephrology and Clinical Immunology, Clinic for Nephrology and Clinical Immunology, University Clinical Centre of Vojvodina, Novi Sad, SRB; 2 Emergency Internal Medicine, Emergency Centre, University Clinical Centre of Vojvodina, Novi Sad, SRB; 3 Allergy and Immunology, Clinic for Nephrology and Clinical Immunology, University Clinical Centre of Vojvodina, Novi Sad, SRB; 4 Nephrology, Clinic for Nephrology and Clinical Immunology, University Clinical Centre of Vojvodina, Novi Sad, SRB

**Keywords:** anti-pl-12 antibody, antisynthetase syndrome, interstitial lung disease, juvenile idiopathic inflammatory myositis, overlap disease

## Abstract

Antisynthetase syndrome (ASTS) is an infrequent autoimmune condition marked by chronic inflammation and the presence of autoantibodies targeting aminoacyl-tRNA synthetases. Clinically, it often manifests through a constellation of features, including inflammatory arthritis, myositis, Raynaud's phenomenon, hyperkeratotic skin changes (mechanic's hands), and interstitial lung disease (ILD). Among these, ILD is the most significant in terms of prognosis, as it contributes to elevated rates of morbidity and mortality, surpassing those seen in other idiopathic inflammatory myopathies.

We report the clinical course of a 56-year-old woman with a prior diagnosis of systemic lupus erythematosus (SLE) established in 2019, based on the 2017 European League Against Rheumatism (EULAR)/American College of Rheumatology (ACR) classification criteria. She was admitted to the Institute for Pulmonary Diseases of Vojvodina (IPBV) in Sremska Kamenica, Serbia, due to respiratory symptoms suggestive of ILD. Although ILD is an uncommon manifestation in SLE, the patient was assessed for the potential coexistence of other connective tissue diseases, such as systemic sclerosis (SSc) and inflammatory myopathies.

Laboratory testing demonstrated a strong antinuclear antibody (ANA) reactivity on Hep-2 cells and low anti-double-stranded DNA (anti-dsDNA) levels (<10 IU/ml). Extended myositis panel testing revealed marked positivity for anti-PL-12 and anti-Ro-52 antibodies.

Given the presence of pre-existing ILD and clinical signs of arthritis and proximal muscle weakness, alongside the identification of anti-tRNA synthetases antibodies, a diagnosis of ASTS was established. Immunosuppressive treatment was intensified by increasing the glucocorticoid (GC) dosage and introducing intravenous cyclophosphamide (CYC, 1 g). The patient demonstrated a favorable clinical and biochemical response to this regimen.

## Introduction

Antisynthetase syndrome (ASTS) represents a rare systemic autoimmune disorder defined by the presence of autoantibodies directed against aminoacyl-tRNA synthetase. The disease encompasses a diverse range of clinical features, notably myositis, arthritis, Raynaud's phenomenon, hyperkeratotic fissured palms ("mechanic's hands"), and interstitial lung disease (ILD). Of these, ILD constitutes the most serious complication, being associated with poor long-term outcomes and a significantly higher risk of mortality than other forms of inflammatory myopathy [1].

First characterized in the early 1980s through the discovery of specific antisynthetase autoantibodies, ASTS was later delineated as a unique clinical entity in the following decade [2]. Among these autoantibodies, anti-Jo-1 is the most frequently encountered, whereas antibodies such as anti-PL-12, targeting alanyl-tRNA synthetase, are considerably rarer, present in under 3% of cases.

The typical clinical phenotype associated with ASTS includes ILD, myositis, arthralgia, and vascular manifestations such as Raynaud's phenomenon. Notably, anti-PL-12 autoantibodies have a particularly strong association with ILD and can present independently of myositis [3]. The current case illustrates this less common presentation, characterized by ILD in the context of anti-PL-12 positivity.

Due to its clinical heterogeneity, ASTS can be diagnostically challenging. The identification of myositis-specific autoantibodies is therefore critical for accurate diagnosis. Moreover, ASTS may overlap with other connective tissue diseases, including SLE, systemic sclerosis (SSc), and rheumatoid arthritis (RA), complicating the clinical picture [4]. We describe a case of ASTS in a patient with pre-existing SLE, focusing on the diagnostic challenges, treatment strategies, and clinical outcome.

## Case presentation

We report the case of a 56-year-old woman diagnosed with systemic SLE in 2019, according to the 2017 European League Against Rheumatism (EULAR)/American College of Rheumatology (ACR) classification criteria, when she was admitted to the Institute for Pulmonary Diseases of Vojvodina (IPBV) in Sremska Kamenica, Serbia, for evaluation due to ILD, diagnosed in the same year, worsening with exertion dyspnea, dry cough, fatigue, low-grade fever, arthralgia, and inspiratory crackles. Although ILD is uncommon in patients with SLE, she was closely monitored for possible overlap with other autoimmune conditions, including SSc and inflammatory myopathy, after symptom progression and the development of a heliotrope rash.

**Figure 1:**
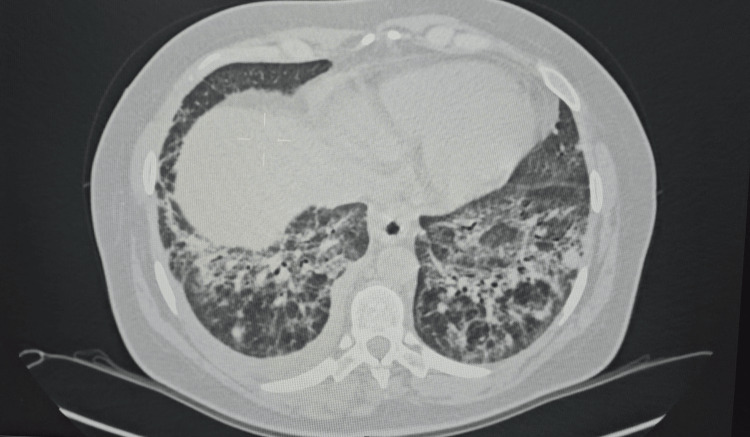
High-resolution computed tomography Parenchymal consolidation with ground-glass opacity diffusely involving the middle lung fields bilaterally and a 6 mm soft-tissue nodule located in segment 6 (S6) of the left lung

Initial therapy included glucocorticoids (GCs), 1 mg/kg/day (60 mg daily), with gradual tapering, and synthetic antimalarials, hydroxychloroquine 200 mg daily. Because of recurrent interstitial pneumonitis, GC doses were escalated, and azathioprine was introduced. However, azathioprine was discontinued after a short period due to the development of a liver lesion. During the June 2020 hospitalization, follow-up high-resolution computed tomography (HRCT) of the chest demonstrated multiple, predominantly peripheral ground-glass opacities (GGO) localized to the right upper lobe and the left anterior and hilar regions, with extensive bilateral involvement of the lower lobe. A 6 mm pulmonary nodule was identified in the left lower lobe, which showed regression six months later with no malignant features (Figure 1).

Spiroplethysmography with diffusing capacity of the lungs for carbon monoxide (DLCO) demonstrated a restrictive ventilatory pattern with DLCO reduced to 59%. Given recurrent disease exacerbations, cyclophosphamide (CYC) was initiated at a dose of 500 mg (12 cycles in total). Between 2020 and 2022, during CYC therapy, the patient remained clinically stable and asymptomatic. Serial laboratory monitoring was unremarkable except for persistently decreased C4 levels, while anti-double-stranded DNA (anti-dsDNA) antibodies stayed low, and SLE activity remained clinically inactive. The initial laboratory findings are presented in Table 1.

**Table 1:** Laboratory investigations HGB: hemoglobin; WBC: white blood cell; RBC: red blood cell; HCT: hematocrit; PLT: platelet; AST: aspartate aminotransferase; ALT: alanine transaminase; GGT: gamma-glutamyl transferase; LDH: lactate dehydrogenase; CRP: C-reactive protein; C3: complement component 3; C4: complement component 4; ESR: erythrocyte sedimentation rate

Test name	Value	Unit	Reference range
HGB	122	g/L	120-160
WBC count	16.1	×10^9^/L	4-10
RBC count	4.69	×10^12^/L	3.9-5.4
HCT	0.384	g/L	0.4-0.5
PLT count	480	×10^9^/L	140-400
Sodium	137	mmol/L	135-148
Potassium	4.1	mmol/L	3.5-5.5
Urea	3.4	mmol/L	2.5-7.5
Creatinine	52	µmol/l	50-115
Uric acid	400	µmol/l	208-428
Bilirubin total	8	µmol/l	3-21
AST	32	U/L	5-37
ALT	25	U/L	5-48
GGT	80	U/L	1-64
LDH	327	U/L	125-220
CRP	79.9	mg/L	0-5
C3	1.46	g/L	0.81-1.8
C4	0.2	g/L	0.3-2.9
ESR	41	mm/h	<15
Fibrinogen	5.46	g/L	1.86-4.86

Cardiac evaluation revealed concentric left ventricular hypertrophy with preserved systolic function (ejection fraction (EF) 65%), mild aortic, mitral, and tricuspid regurgitation, and an estimated right ventricular systolic pressure (RVSP) of 33 mmHg. Spirometry was normal, with DLCO slightly reduced (64%). After the completion of 12 CYC cycles, maintenance therapy with mycophenolate mofetil (MMF) and hydroxychloroquine was introduced, while GCs were tapered.

After one year, the patient's condition deteriorated, presenting with subfebrile temperature, dry cough, fatigue, arthralgia, and inspiratory crackles. Dermatological findings included a heliotrope rash of the eyelids and erythematous lesions on the upper arms and back.

Repeated immunoserological testing revealed antinuclear antibody (ANA) positivity on Hep-2 cells with an intensely positive dot pattern and anti-dsDNA <10 IU/ml. Myositis profile 2 demonstrated strong reactivity for PL-12 (+++) and Ro-52 (+++). HRCT of the lungs and echocardiography showed stable findings without evidence of right heart involvement. Expert review for granulomatous diseases and ILDs determined that antifibrotic therapy with pirfenidone was not indicated.

Electromyoneurography (EMNG) confirmed myopathic changes. Considering the pre-existing ILD, arthritis, muscle weakness, and the presence of anti-tRNA synthetase antibodies, the patient was diagnosed with ASTS. Immunosuppressive therapy was intensified by escalating GC dosage and initiating intravenous CYC (1 g), based on the EULAR recommendations (0.5-1 g/m^2^ every four weeks). The patient demonstrated a rapid clinical and laboratory improvement following treatment, with the resolution of subjective symptoms and also with the normalization of inflammatory markers (C-reactive protein). During follow-up, there was significant clinical improvement, with the complete resolution of pulmonary symptoms, normalization of muscle enzyme levels, and stabilization of both HRCT findings and pulmonary function tests. The patient has remained stable on maintenance therapy with intravenous CYC and low-dose prednisone. Throughout the observation period, there has been no evidence of relapse of ILD or myopathy.

## Discussion

This case underscores the diagnostic complexity of ASTS, particularly when its presentation overlaps with SLE. The gradual evolution of symptoms highlights the importance of maintaining a high index of suspicion in patients with multisystem involvement.

The diagnosis of ASTS is inherently difficult due to its resemblance to other autoimmune conditions, such as RA and SSc. Nevertheless, a possible explanation could be its association with SLE. In this patient, recognition was achieved through clinical evaluation combined with serological testing for myositis-specific autoantibodies, which proved critical for initiating timely and targeted therapy. Current classification systems, including the 2017 EULAR/ACR criteria for idiopathic inflammatory myopathies (IIM), do not formally define ASTS as a separate entity. Among myositis-specific autoantibodies, anti-Jo-1 remains the only marker incorporated into these criteria, most likely due to the limited representation of other antibodies in the original datasets [2].

ILD represents the most frequent systemic manifestation of ASTS, with prevalence estimates ranging from 67% to nearly 100%. The clinical course may be subtle, beginning with a persistent dry cough or exertional dyspnea, although asymptomatic cases are not uncommon and may be detected incidentally, which proves the importance of CT diagnostics in these patients. Patients with anti-PL-7 or anti-PL-12 antibodies are more likely to develop ILD and often experience a more aggressive, rapidly progressive phenotype compared to those with anti-Jo-1 antibodies [4]. Pulmonary function tests (PFTs) typically reveal a restrictive pattern, including reduced forced vital capacity (FVC), total lung capacity (TLC), and DLCO. HRCT, the gold standard for evaluating ILD, frequently demonstrates patterns such as nonspecific interstitial pneumonia (NSIP), organizing pneumonia (OP), or NSIP/OP overlap, while usual interstitial pneumonia (UIP) and acute interstitial pneumonia are reported less frequently [5-7].

The detection of anti-Ro-52 antibodies in our patient carries clinical significance. These antibodies are associated with insidious disease onset and accelerated ILD progression, often translating into a poorer prognosis. Their coexistence with anti-Jo-1 has also been linked to increased malignancy risk, particularly colorectal and gynecologic cancers such as ovarian and breast cancer [7]. In contrast, a 2021 meta-analysis suggested that the presence of antisynthetase-related autoantibodies may be associated with a lower overall cancer risk compared to other forms of inflammatory myopathy, though available data remain limited [8]. Current initiatives by the International Myositis Assessment and Clinical Studies Group aim to establish standardized cancer risk assessment and screening guidelines for patients with inflammatory myopathies. Although no malignancy was detected in our patient, ongoing surveillance remains prudent.

Treatment of ASTS-associated ILD continues to pose challenges due to the absence of randomized controlled trials. Current management strategies are extrapolated from protocols designed for other myositis-related ILDs [1]. GCs remain the first-line therapy for both pulmonary and muscular involvement, though consensus regarding dosing and tapering regimens is lacking. To minimize long-term steroid exposure, additional immunosuppressive agents such as MMF or azathioprine are often introduced early. CYC is typically reserved for severe ILD, while rituximab is increasingly employed as salvage therapy in refractory cases [4].

In summary, this case illustrates the need for heightened clinical vigilance when evaluating patients with overlapping autoimmune features and respiratory involvement. Comprehensive antibody profiling, timely recognition, and early initiation of appropriate immunosuppressive therapy are crucial to improving long-term outcomes in ASTS.

## Conclusions

The heterogeneous presentation of ASTS and its overlap with other rheumatic diseases highlight the need for a multidisciplinary approach involving rheumatologists, pulmonologists, and immunologists. Early recognition and timely initiation of immunosuppressive therapy are essential to control inflammation, limit disease progression, and improve patient outcomes and quality of life.
